# iPSC Bioprinting: Where are We at?

**DOI:** 10.3390/ma12152453

**Published:** 2019-08-01

**Authors:** Sara Romanazzo, Stephanie Nemec, Iman Roohani

**Affiliations:** 1 Biomaterials Design and Tissue Engineering Lab, School of Chemistry, University of New South Wales, New South Wales 2052, Australia; 2 School of Materials Science and Engineering, University of New South Wales, New South Wales 2052, Australia

**Keywords:** 3D bioprinting, iPSC, tissue engineering, pluripotent stem cells, biofabrication

## Abstract

Here, we present a concise review of current 3D bioprinting technologies applied to induced pluripotent stem cells (iPSC). iPSC have recently received a great deal of attention from the scientific and clinical communities for their unique properties, which include abundant adult cell sources, ability to indefinitely self-renew and differentiate into any tissue of the body. Bioprinting of iPSC and iPSC derived cells combined with natural or synthetic biomaterials to fabricate tissue mimicked constructs, has emerged as a technology that might revolutionize regenerative medicine and patient-specific treatment. This review covers the advantages and disadvantages of bioprinting techniques, influence of bioprinting parameters and printing condition on cell viability, and commonly used iPSC sources, and bioinks. A clear distinction is made for bioprinting techniques used for iPSC at their undifferentiated stage or when used as adult stem cells or terminally differentiated cells. This review presents state of the art data obtained from major searching engines, including Pubmed/MEDLINE, Google Scholar, and Scopus, concerning iPSC generation, undifferentiated iPSC, iPSC bioprinting, bioprinting techniques, cartilage, bone, heart, neural tissue, skin, and hepatic tissue cells derived from iPSC.

## 1. Introduction

Tissue engineering has been widely studied for the repair and regeneration of a variety of body tissues, such as cartilage, bone, heart, skin, and neural tissue. The design of a bioengineered construct, including the choice of materials and biological components, is critical for its biofuntion [1].

Common stem cells used in tissue engineering applications can be classified based on their origin as embryonic stem cells (ES), induced pluripotent stem cells (iPSC), and adult stem cells. ES are pluripotent stem cells derived from the inner cell mass of an early-stage embryo [2]. They terminally differentiate in vitro into differentiated cells, such as cardiomyocytes (CM) and thus are considered a robust cell model for in vitro tissue regeneration [3]. About a decade ago, a new cell type was developed by Prof. Yamanaka, known as iPSC, which showed similar characteristics of ES. iPSC were first generated from mouse fibroblasts by the introduction of four transcription factors (Oct4, Sox-2, c-Myc, and Klf-4) through genetic reprogramming [4]. Subsequently, iPSC were generated from other adult cells (e.g., neural cells, keratinocytes, renal cells, adipose stem cells) through different methods, which will be described in this review [5,6]. The primary advantages of using iPSC include: (i) self-renewal at a large scale, unlike adult stem cells, (ii) pluripotency, they can give rise to all cell types, (iii) autologous source, can be generated from terminally differentiated cells taken from patients with a non-invasive method, and thus overcome the immune rejection issue related to ES use, (iv) do not raise ethical issues compared to ES, and (v) having a comparable differentiation ability to ES. The transcriptional profiles of iPSC and ES are nearly identical, proven by utilizing iPSC in the production of live-born and fertile animals via tetraploid complementation. Furthermore, human iPSC can differentiate in vitro into diverse lineages, including cardiomyocytes, neurons, hematopoietic progenitors, endothelial cells, osteoclasts, hepatocyte-like cells, islet-like cells, and retina [7].

**Figure 1:**
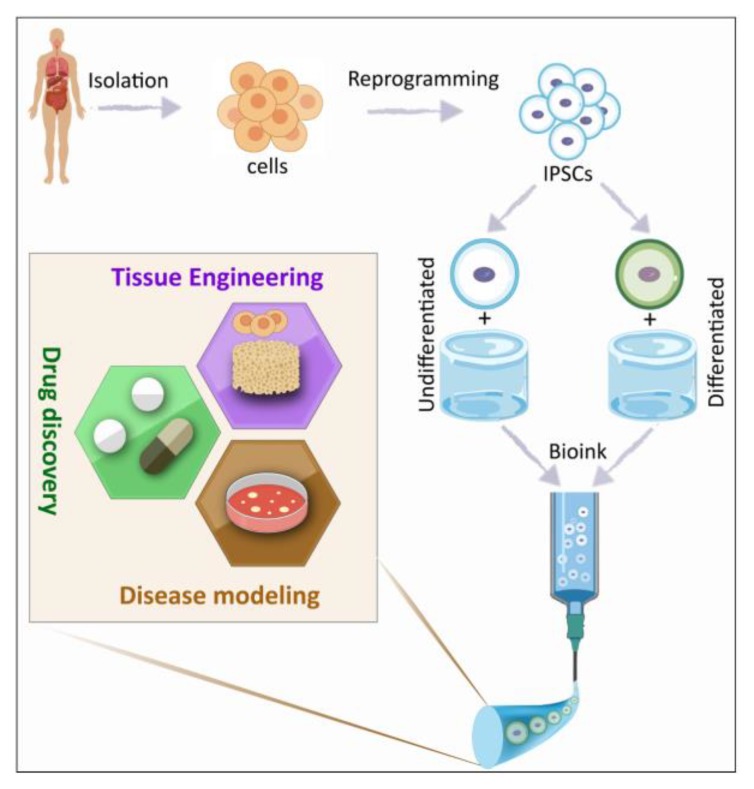
Potential of induced pluripotent stem cells (iPSC) for regenerative medicine, disease modeling, and drug discovery.

Although many challenges remain unaddressed for iPSC technology, such as the tendency for tumors to evolve after iPSC transplantation and the low efficiency of their generation technology, iPSC are entirely changing how the treatment of diseases are approached in biomedical research. Currently, iPSC are not only used in regenerative medicine applications but also for disease modeling and drug discovery (Figure 1). Moreover, stem cell companies are using iPSC derived stem cells for a variety of clinical trials and have proven the potential of this cell source for clinical application [8].

Over the past three decades, 3D bioprinting has evolved as a tool to create multiscale architectures with accurately positioned cells and biomolecules. This technology enables one to mimic the complexity of native tissues or organs, which is known to be crucial to recapitulate microarchitecture of specific tissues and promotes the tissue engineering purpose of replacing or regenerating damaged and diseased tissues. Various sources of iPSC and types of biomaterials are used in combination with bioprinting techniques. Typically, cells are combined with natural or synthetic biomaterials to form bioinks [9], which are used to fabricate scaffold-based or scaffold-free constructs. This review recapitulates the current technologies of bioprinting for iPSC at the undifferentiated stage, the influence of printing parameters and condition on cellular function, and composition of available bioinks. We will further discuss the advantages and disadvantages of bioprinting techniques, different sources of iPSC and bioinks. 

## 2. iPSC Generation

The scientific community has recognized the profound change of landscape that the discovery of iPSC has imbued on the field of biomedical research [10,11,12,13]. Following Prof. Yamanaka’s first iPSC generation, scientists around the world have focused on finding an efficient and less invasive method (e.g. the level of vector sequences integrated into iPSC genome) to reprogram adult somatic cells into a pluripotent stage by introducing transcription factors using a delivery vector. 

**Figure 2:**
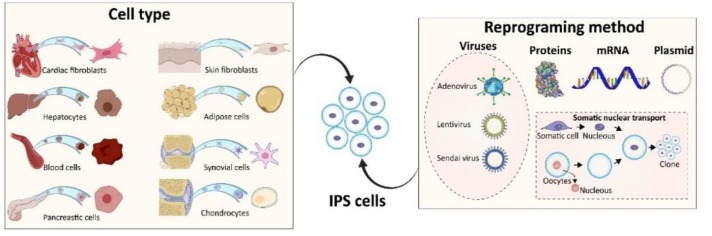
Schematic of iPSC generation process. A variety of cell sources (**left**) can be used to generate iPSC, through different methods (**right**).

In the first successful iPSC reprogramming method, a retrovirus was used to transduce cells for the overexpression of four transcription factors: Oct4, Sox2, Klf4, and c-Myc, to stably convert adult cells to a pluripotent state that can be maintained and replicated through cell mitosis. The four factors were chosen based on their role in maintaining cell pluripotency, defined as the ability to generate all cell types of the body. Starting transgene expression efficiency for nuclear reprogramming was close to 0.02% at ~30 days after transducing the four factors [14], whereas, the highest reprogramming efficacy so far reported to date is at about 1.5%. Existing iPSC generation techniques can be divided into four main groups (Figure 2), which we will discuss here. 

### 2.1. Virus-Based Methods

Various types of viruses have been used as reprogramming vectors, divided based on their ability to integrate into the reprogramming cell’s genome. Lentivirus, an integrating vector, has achieved a good reprogramming efficiency ranging from 0.1% to 1.5%. However, the main disadvantage of using the lentiviral vector is the incorporation of vector sequences into the iPSC genome. This integration could promote oncogenesis by altering local gene expression and hence hampering translational opportunity to the therapeutic application [15]. On the contrary, adenovirus is a non-integrating virus, and thus, considered an appealing source for delivering the factors needed for iPSC generation. However, a significantly low reprogramming efficiency (0.0002% in human cells) has lowered the popularity of using this virus as a reprogramming method. Sendai virus is an RNA virus that does not enter the nucleus. Thus, it can be cleared out from the cells after around ten passages. Highest efficiency reported for reprogramming with Sendai virus after 25 days from the transduction, is 0.1% for blood cells, such as T cells and CD34 (+) cord blood cells, and 1% for fibroblasts. Sendai virus also has the ability to produce a large number of proteins and thus improve reprogramming efficiency [11]. Overall, most of the virus delivery methods have the associated risk of chromosomal instability and tumorigenesis, due to the inserted mutagenesis of the viral vector. 

### 2.2. Proteins

The use of reprogramming factors as expressed proteins is favorable, as it is the least invasive vector for iPSC generation. However, some studies have shown that proteins of the four transcription factors, Oct4, Sox2, Klf4, and c-Myc, obtained by E. Coli transformation, yield an iPSC generation efficiency up to 0.001% in human fibroblasts [16,17,18]. This low-efficiency is mainly due to difficulties in delivering proteins intracellularly, because of the large size of proteins and the hydrophobic property of cellular membrane. 

### 2.3. Somatic Cell Nuclear Transfer (SCNT)

SCNT is a technique where a somatic cell nucleus is fused with a mature enucleated oocyte that might result in 100% reprogramming efficiency [19]. However, ethical and technical issues related to the process refrain the competency of this method.

### 2.4. Other Methods

Other methods of iPSC generation include reprogramming factors expressed as messenger RNA (mRNA) that when added to the adult cell of interest can be reverted to its pluripotent state. This approach has yielded the highest reprogramming efficiency obtained so far, 1.4%. However, it is not a commonly used approach, developed solely for fibroblasts, due to its laborious procedure that requires the addition of mRNA for seven consecutive days. All methodologies for iPSC generation have been extensively described by Malik et al. [11].

### 2.5. iPSC Cell Source

Fibroblasts are the most commonly used cell type to generate iPSC because of the high availably and access via isolation from the skin tissue. Other cell types used for the generation of iPSC are cells isolated from blood, urine, pancreatic islet beta cells, synovial cells, and mesenchymal stromal cells from wisdom teeth [20]. Several studies have shown that stem cells or progenitor cells in tissues such as muscle, blood, liver, skin, and brain, are more responsive in being reprogrammed compared to other terminally differentiated cells [21]. Moreover, it has been shown that iPSC at early stage passages resemble DNA methylation characteristics of the originated cell type, suggesting the importance of cell source for the suitability of iPSC based on the ultimate application [22]. The cell sources and their reprogramming methodologies into iPSC, are illustrated in Figure 2. 

## 3. 3D Bioprinting Techniques

3D bioprinting is defined as a layer-by-layer placement of cell-laden bioinks, with spatial control of functional components, to fabricate 3D structures [23]. To briefly describe the process, first, physiologically relevant shapes are generated using computer-aided design (CAD). The CAD model is digitally cut into 2D slices, then converted into a numerical control programming language (G-code) that instructs the printer’s spatial coordinates and injection volume [24]. Both scaffold-based and scaffold-free methods have been used to bioprint numerous cell types (cardiac, cartilage, dermal, hepatic, neural) as enumerated in this review.

### 3.1. Biomaterials

A variety of biomaterials, made from natural or synthetic materials or a combination of the two as hybrid materials, have been used for bioprinting [25,26]. They can be mainly divided into hydrogels, microcarriers, and decellularized matrix components. 

Hydrogels are the most commonly used materials for 3D bioprinting such as alginate, agarose, chitosan, fibrin, gelatin, hyaluronic acid (HA) and Matrigel, and Pluronic® F-127 and poly(ethylene glycol) (PEG) [9]. A suitable hydrogel for bioprinting should have an elastic modulus ranging from 10^2^–10^3^ Pa and a viscosity of 30 mPa/s to 6 × 10^7^ mPa, to be suitable for extrusion bioprinting [27]. Hydrogels often need a crosslinker, during or immediately after the bioprinting to form the final shape of the intended tissue constructs. Natural polymers can be further divided in protein-based, polysaccharides, and extracellular matrix (ECM) derived hydrogels. 

Synthetic polymers such as PEG and its derivates (PEGDA, PEGX-PEG, PEGX-gelatin, PEGX-gelatin-fibrinogen, and PEGX-gelatin-atelocollagen), offer strong mechanical properties facilitating shape maintenance of printed construct during the bioprinting process [9]. Pluronic is generally used in bioprinting for producing sacrificial structures. It has excellent printability and can be cross-linked by increasing the temperature. Pluronic can be easily washed away after printing if needed, as it turns to liquid at 4 °C or lower temperatures.

Microcarriers are small polymeric beads made from dextran, gelating or cellulose, which are mostly used as support materials for bioprinting. Generally, hydrogels are mechanically weak and cannot sustain physiological loads from body tissues, such as bone, cartilage, and tendon; thus, they often needed to be combined with stiff biomaterials. For example, alginate is often mixed with gelatin, or cellulose, agarose with collagen, chitosan with gelatin, and cellulose with hyaluronan [28]. Hydrogels can be combined with a variety of growth factors to induce specific tissue formation such as vasculogenesis, which is required for almost all types of body tissues. Faramarzi et al. developed a bioink based on alginate and patient-specific platelet-rich plasma (PRP), which is known to have a high content of angiogenic factors and thus useful for tissue regeneration [29].

### 3.2. 3D Bioprinting Strategies

**Figure 3:**
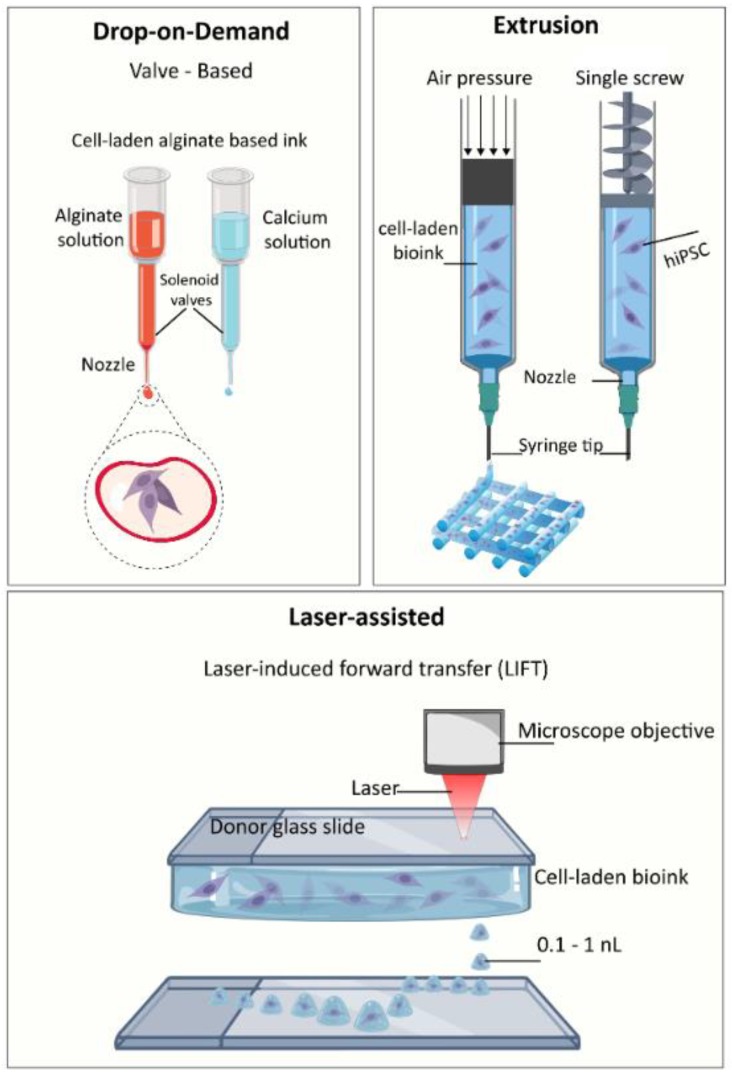
Schematic representation of printing techniques used for iPSC bioprinting.

The bioprinting techniques can be classified into three main modalities: (i) inkjet/droplet, (ii) microextrusion, and (iii) laser-assisted printing [9,27,28]. Each technique manipulates the cell-hydrogel bioink to precisely articulated positions, generating 3D structures for the cells to continue to proliferate in. Figure 3 shows a schematic representation of bioprinting techniques.

In inkjet/droplet-based techniques, thermal, piezoelectric, or electromagnetic forces expel drops of bioink on a substrate, in a high-throughput manner. Drops can be formed either by continuous inkjet printing or drop-on-demand. Typical cell viability obtained with this technique ranges from 80% to 95%. Drop-on-demand generates drop volumes on the order of 1–100 pL. Inkjet printing, in general, is fast and low-cost; and bioinks should have a low viscosity to avoid clogging the nozzle [30].

Extrusion-based bioprinting is a technique that uses pneumatic, piston, or screw force to push viscous cell-ink solutions out of a tip [31]. Extrusion allows for printing of highly viscous, cell-dense bioinks; however, cells experience high shear stresses by moving through the tip. Finally, laser techniques include laser-assisted bioprinting based on the laser-induced forward-transfer (LIFT) where a pulsed laser forms a bubble that transfers the ink to an absorbing layer below [32] and stereolithography (SLA) that patterns photosensitive solutions by light exposure [33]. SLA is sometimes considered a separate technique because of its need for photopolymerization [34]. It is a quick and accurate method, but with a limited choice of biomaterials and requiring an intense-ultraviolet (UV) exposure. Laser-assisted printing offers a high resolution and precision; however, it is costly and labor-intensive with unpredictable laser effects on biological components. Typically, laser-assisted bioprinting yields higher cell viability (>90%) compared to SLA, inkjet, and extrusion with less than 80% viability. 

Needle-based bioprinting techniques are more popular than other methods due to accessibility and ease of using Luer-lock syringe tips [35,36]. However, needle tips are prone to clogging, especially at diameters under 150 μm [37]. In bioprinting, the common factors that appear to impact cell viability are shear stress, laser exposure, heat, and vibration [38,39]. Selecting a bioprinting technique ultimately depends on the application, such as disease modeling, drug screening, and tissue or organ engineering. Design factors in bioprinting include shape and resolution, material heterogeneity, and cellular-material remodelling dynamism, which are used in bioprinting strategies, defined by Lee et al. as direct bioprinting, in-process crosslinking, post-process crosslinking, indirect bioprinting and hybrid bioprinting. In direct bioprinting, materials are printed directly to form the pre-determined configurations. In-process crosslinking, instead, is achieved either by co-extrusion of bioink and crosslinkers or by sequentially depositing the two components.

On the contrary, post-process crosslinking requires a mixture of materials with multiple crosslinking mechanisms. Indirect bioprinting consists of printing the bioink together with a support material, which is removed from the construct through post-processing. Hybrid bioprinting aims at combining printing techniques with other fabrication processes, such as electrospinning and melt-potting [40]. Various quantitative measures can inform bioprinter selection such as cellular viability before, during, and after printing [41], gene expression compared to 2D cultures [42], and structural confirmation via fluorescent, dark field, bright field confocal imaging [43]. The current challenge facing the field includes leveraging non-invasive imaging techniques such as Magnetic Resonance Imaging (MRI), Positron Emission Tomography (PET), X-ray Computed Tomography (CT) techniques, and ultimately developing methods tracking live images of the printed and in vitro structures to obtain individual-print specific and aggregate cellular, mechanical, and tissue response [40]. 

In addition to the scaffold-based bioprinting approach, recently few of the so-called “scaffold-free” printing techniques have emerged, which consists in the use of cell or cell aggregates, without any support material for bioprinting process. The main advantage of this method is the biocompatibility of the 3D printed construct [44], as it provides a natural environment for cells and brings better cell-cell interaction, compared to bioink based 3D printing techniques. These features are well-known to be critical for iPSC viability. Existing scaffold-free systems can be classified according to the type of building blocks used (cell sheets, isolated single cells, or spheroid cell aggregates) or the processes involved in the formation of artificial tissues or building blocks [45]. An example of scaffold-free bioprinting method was given by Bakirci et al. in 2017, who first printed cell aggregates obtained by the formation of cell-sheet. Briefly, human skin fibroblasts were cultured on poly(N-isopropylacrylamide) coated dishes and by temperature switch from 37 °C during cell culture, to 24 °C, a cell-sheet was lifted from the dish and used to prepare cell aggregates to be printed with a syringe based extrusion Novogen MMX (Organovo) bioprinter [46]. A bioprinter with two printheads was used to co-print a layer of 2% w/v agarose, to support the layer of cell aggregates and the second print head to build the construct layer-by-layer. iPSC has already been used in few studies for the fabrication of 3D printed constructs, mainly for tissue engineering applications. The pluripotent stem cells have either been utilized in an undifferentiated state or induced into a specific pathway of differentiation, and then used for printing. In the following section, we will discuss bioprinting of undifferentiated iPSC and differentiated iPSC.

## 4. Bioprinting Undifferentiated iPSC

The main obstacle in bioprinting undifferentiated iPSC is their sensitivity to mechanical forces during the printing process [47,48,49]. Therefore, bioprinting parameters should be carefully optimized for the bioprinting of undifferentiated iPSC. Depending on the printing technique, cells experience high shear forces, radiation (laser), and electric or thermal stresses. iPSC bioprinting requires aggregates of differentiated embryoid bodies (EB) embedded in hydrogel bioinks [37]. Maintenance of EB aggregates is crucial for iPSC pluripotency and proliferation [50]. Additionally, iPSC-laden bioinks undergo ionic, chemical, temperature, and light stress during crosslinking. Current bioprinting techniques of undifferentiated iPSC are drop-on-demand, extrusion, and laser-assisted methodologies. Faulkner-Jones et al. used the valve-based bioprinting technique for printing human embryonic stem cells and iPSC in 2015 [51]. Reid et al. developed a precise and affordable technique to print a single cell per injection [37]. Gu et al. printed iPSC in a polysaccharide-based bioink [52], Nguyen et al. co-printed iPSC with irradiated chondrocytes, and Li et al. enhanced post-printed survival with a chitin based bioink [53]. Koch et al. printed iPSC using laser-assisted printing with a neodymium-doped yttrium aluminum (Nd:YAG) 1064 nm laser [54]. Table 1 shows the reports on the bioprinting of undifferentiated iPSC in the literature and highlights the technique and bioprinting parameters.

### 4.1. Bioprinting Techniques and Nozzle Diameters

Gu et al. and Nguyen et al. selected commercially available pressure extrusion bioprinters: “3D Bioplotter” and “3D Discover”, respectively. Reid et al. attached glass needle tips to a commercially available plastic 3D printer (Felix 3.0, Isselstein, The Netherlands) and Li et al. custom-built a screw extrusion bioprinter. Faulkner-Jones et al. leveraged a valve-based system and nozzle from Lee Products Ltd. The nozzle tips used among these studies had a diameter ranged from 40 µm glass tips (Reid) to 300 µm syringe tip (Nguyen). Gu et al. and Li et al. used nozzles with a diameter of 200 and 260 µm, respectively. Li et al. tested needles with a range of diameter (160, 210, 260, 310, 360 μm) and extrusion rates (2, 3, 4, 5, 6 mm/s), and found the 260 μm at 5 mm/s resulted in the best survival rates (over 90%). Faulkner-Jones et al. used a nozzle with an internal diameter of 101.6 µm with two different lengths, one of 8.9 and the other of 24.4 mm. When the viability of cells assessed, a shorter nozzle resulted in higher cell viability (more than 84%), whereas cell viability after using the longer nozzle was ~ 71%. The correspondence of cell viability to the length of the nozzle is attributed to an increased exposure time of cells to shear stress forces through nozzle [52,53,55,56]. 

### 4.2. Bioinks and Crosslinkers

A variety of hydrogels have been used as bioink for the printing of undifferentiated iPSC. Alginate, a naturally derived and biocompatible polysaccharide, is commonly used as the bioink due to its facile cross-linking via calcium ions [57]. Faulkner-Jones et al. developed a 1.5% w/v alginate solution bioink for their valve-based bioprinter [51]. The addition of other polysaccharides can mechanically improve bioink formulations of alginate and helo to encapsulate cell spheroids [58]. Gu et al. combined 5% w/v alginate, 5% w/v carboxymethyl-chitosan, 1.5% w/v agarose to prepare an extrudable and porous ink. Nguyen et al. investigated nanofibrillated cellulose (NFC) with HA and alginate (A) and found the best iPSC viability in NFC/A ratio of 60/40. Li et al. formulated a thermoresponsive 2% w/v hydroxypropyl chitin (HPCH) bioink functionalized with 0%–30% Matrigel, a solubilized basement membrane extract from murine Engelbreth-Holm-Swarm mouse sarcoma (Corning). Reid et al. used Geltrex (Thermo Fisher), another solubilized basement membrane extract and media. Koch et al. investigated various ink formulations with laser-assisted bioprinting: Matrigel, Geltrex, alginate, collagen type I from rat tail, HA, and fibrinogen with thrombin. The best cell viability was observed for a bioink with 1 wt % HA in medium or fibrinogen printed onto a Matrigel [37,52,53,54,55]. In terms of crosslinkers, alginate bioinks require calcium ions, and HPCH solidifies by temperature (37 °C). Current crosslinking methods of bioinks with undifferentiated iPSC have been limited to metallic ions and temperature, as they are less stressful to cells compared to other crosslinking methods such as UV, enzymatic, and polymeric crosslinkers. Nevertheless, such crosslinkers are more effective in generating intricate structures and preventing bioink from spreading after printing. There are; however, strategies to enhance the print resolution even by using less effective crosslinkers such as using a slurry of particles to form a support bath, to lightly cross-link and hold the ink to retain its shape during printing [59].

## 5. Bioprinting iPSC Differentiated iPSC 

Researchers have successfully generated bioconstructs through a variety of bioprinting techniques to repair cartilage, bone, cardiac, nervous, liver, and vascular tissues [23,60,61]. Recently bioprinting of adult stem cells and terminally differentiated iPSC derived cells has attracted much attention and are growing rapidly. The key interest is the potential of iPSC to self-renew and their ability to differentiate into all cell types of human tissues. However, the limiting factor in clinical translation is that printed iPSC constructs are unable to form viable and vascularized tissue. 

### 5.1. Cartilage and Bone

Articular cartilage damage has a limited healing potential, forming fibrous scar tissue with compromised mechanical and biochemical properties. Bioprinting has emerged as a promising technology to address this, by the creation of organized and living constructs layer-by-layer to mimic natural cartilage or the osteochondral interface [62]. Current treatment strategy includes autologous chondrocytes implantation (ACI), which requires two invasive surgical procedures, and healing depends on autologous chondrocytes quality and quantity [63]. Moreover, ACI has failed to show a significant clinical outcome when compared to other techniques, such as microfracture, particularly in the reconstruction of large defect units [64]. Since cartilage has low immunogenicity, heterologous cells can be utilized in 3D bioprinting technologies to help recreate the complex architecture of the native cartilage structure in vitro [65]. Some of the bioinks used in cartilage bioprinting are approved by FDA. The first porcine collagen type I/III scaffold ACI (matrix-induced autologous chondrocyte implantation; MACI) was FDA approved in December of 2016. The successful outcome of MACI clinical trials promises a bright future for the clinical translation of bioprinted scaffolds. There have been promising in vitro and in vivo studies looking at 3D bioprinting of engineered cartilage tissue [34]. Thus far, extrusion-based bioprinting using alginate and scaffold-free bioinks have resulted in the best outcome for cartilage regeneration [61,66]. Furthermore, as an example of intraoperative repair technique, Di Bella et al. performed printing in situ in a sheep model with the weight-bearing surface of the lateral and medial condyles of both femurs. Hydrogel composed of gelatin methacrylamide (GelMA) and hyaluronic acid methacrylate (HAMA) was UV-crosslinked right after deposition and was able to repair the defects Di Bella et al. [67]. This technique can avoid usage of pre-printed bench-based tissue engineering, but no human study has been conducted so far. Cell-laden hydrogels have also been investigated as bioink for 3D bioprinting of cartilage biomimetic structures [68]. The combination of such bioink with solid 3D printed scaffolds made from synthetic polymers, such as polycaprolactone or Pluronic F127, has also been tested for cartilage repair [69,70]. The team of researchers at Chalmers University of Technology, successfully created a cartilage tissue by 3D bioprinting of iPSC derived from cells taken from patients undergoing knee surgery [55]. This is the only study, thus far, to report bioprinting of iPSC derived cells for cartilage tissue engineering applications. Other methodologies differentiate iPSC into chondrocytes, such as the co-culture of iPSC with primary chondrocytes, the preparation of embryoid bodies (EB) iPSC, followed by the differentiation of the mesodermal cells in the EBs into chondrocytes by treatment with growth factors, the differentiation of iPSC into mesenchymal stem cell-like cells, followed by their differentiation into chondrocytes by chondrogenic supplementation [71,72,73]. Similarly, iPSC can differentiate into osteoblast-like cells, through different methods [74,75]. Studies have been mainly focused on the differentiation of iPSC into osteogenic progenitor stem cells, showing that the MSC derived from iPSC have a more identical gene expression profile to BM-MSC, compared to ES derived MSC. Gao et al. showed an improved bone and cartilage tissue formation using 3D inkjet system to print MSC embedded in PEG-GelMA hydrogel [76]. In a study, Kelly’s lab in Trinity College Dublin, combined a biocompatible ink, made of a gamma irradiated alginate gel with Arg-Gly-Asp (RGD) adhesion peptides, embedded BM-MSC, co-printed with ε-polycaprolactone in order to mechanically reinforce the 3D construct, which was then implanted subcutaneously into the back of nude mice. After 12 weeks from implantation, the construct developed vascularized, mineralized bone with trabecular-like endochondral bone [77]. Other types of bioinks, such as extracellular matrix (ECM) derived or pre-printed scaffolds, composed of hydroxyapatite, showed good osteogenic capability when augmented with BM-MSC [65,78]. No study so far has reported using iPSC and 3D bioprinting for bone tissue engineering; however, different research groups are working on the application of iPSC for osteogenic differentiation and iPSC derived MSC for bone repair purposes. Prof. Xu at University of Maryland and his team have developed iPSC derived MSC (iPSC-MSC) with a genetic modification for the expression of bone morphogenetic protein 2 (BMP2) and combined with calcium phosphate-based scaffolds, which is known to be both an osteoconductive and osteoinductive material, and thus an ideal material for bone regeneration [79]. They observed overexpression of BMP2 in iPSC-MSC, which enhanced osteogenic differentiation of these cells, when seeded on RGD, modified calcium phosphate scaffolds.

### 5.2. Heart

Traditional tissue engineering strategies have not yet been able to address all the requirements to build functional cardiac tissues [78]. Challenges in cardiac tissue engineering are related to cell adhesion and alignment, electric impulse, vascularization, the thickness of cardiac constructs, and tissue integration [78]. 3D bioprinting facilitates an alternative strategy to develop heterogeneous 3D constructs, with appropriate mechanical and biological properties that can rapidly integrate with native tissues [80]. It has been demonstrated that hiPSC differentiate into functional CM representing an appealing cell source for clinical applications. When allogenic iPSC-derived cardiomyocytes (iPSC-CM), generated from fibroblasts, were transplanted into infarcted model in a cynomolgus monkey, they were able to regenerate primate heart tissue [81]. Techniques including scaffold-free and scaffold based, have successfully generated 3D cardiac engineered constructs [82], which are summarized in detail in Table 2. Among the scaffold-free 3D printing techniques, Arai et al. printed cell spheroids onto a needle array creating a tubular cardiac construct, which functioned as a cardiac pump [82]. iPSC-CM, human umbilical vein endothelial cells (HUVEC), normal human dermal fibroblasts (NHDF) were mixed and seeded in low attachment plates, in order to form these cardiac spheroids. Subsequently, spheroids were printed layer-by-layer through a needle array, and after seven days transferred to a bioreactor system, where they were cultured for another seven days. As a result, contraction, spheroid fusion, and cellular reorganization were observed in a comparable way to donor tissues. Similarly, Ong et al. 3D printed cell spheroids with similar composition, forming cardiac patches that were then implanted. The patches spontaneously beat after printing. Moreover, implantation showed engraftment in the native rat myocardium and vascularization [60].

Regarding scaffold-based 3D constructs, most studies have used pre-fabricated 3D scaffolds before adding iPSC-CM or cardiac progenitor cells. Gao et al. performed a study where a 3D printed scaffold made of GelMA was cultured with CM, smooth muscle cells (SMCs), and endothelial cells (ECs) all differentiated from hiPSC derived from cardiac fibroblasts. The same group previously demonstrated iPSC generated from cardiac fibroblast are a better cell source compared to skin fibroblasts, since they seem to be more effective for treatment of myocardial injury and their Ca^2+^ handling profile is more cardiac-like compared to the skin fibroblasts derived iPSC [83]. 

Zimmerman’s lab has recently developed an engineered heart muscle (EHM) by pre-printing a holder, where collagen (ECM derived biomaterial) fibroblasts and iPSC-CM were cultured [84]. 3D printing techniques have been used to build a 3D microphysiological platform with cardiac organoids formed of iPSC-CM [85]. A few other studies of iPSC-CM show the suitability of the cell source not only for drug development and disease modeling, but for the regeneration of heart tissues. Bursac’s group created cardiac patches, composed of a hydrogel solution of human fibrinogen, Matrigel, and thrombin, where iPSC-CM were cultured and then implanted onto rat hearts. These cardio patches showed to engraft solidly and maintained their electrical function, without increasing the arrythmias occurrence [86]. Maiullari et al., who pioneered co-printing iPSC-CM and HUVEC, encapsulated cells in alginate and PEG-fibrinogen hydrogel, using extrusion technique with a custom microfluidic printing head (MPH) [87]. The ability of printing defined geometries and blood vessel-like shapes lead to the grafting of the 3D constructs when implanted subcutaneously in NOD-SCID mice. A very recent paper from Noor et al. reported printing cardiac patches, composed of cells and bioink derived from the same biopsy from the patient. An omental tissue biopsy was used both to extract omental stromal cells and to develop a decellularized ECM bioink. The omental cells were then reprogrammed into iPSC and subsequently differentiated into either CM or EC. Through the novel technology of printing in support medium, composed of sodium alginate, xanthan gum and calcium carbonate, Noor et al. were able to print functional vascularized patches modeled after the patient’s anatomy [88]. 

### 5.3. Hepatic Tissue 

Depending on the severity of liver damage (following acute liver disorder, inherited disease, or chronic liver disease), treatment varies from the direct infusion of hepatocytes, implantation of 3D engineered constructs, to organ transplantation. Several studies propose 3D printed constructs, by combining a variety of natural (e.g., alginate, cellulose, decellularized ECM) and synthetic materials (e.g., polylactide-co-glycolide, polyethylene glycol, and polycaprolactone), with immortalized cell lines or stem cells [89]. Ma et al. successfully printed hiPSC derived hepatic progenitor cells (hiPSC-HPCs), obtained from in vitro differentiation of iPSC generated from skin fibroblast. hiPSC-HPCs were embedded in a solution of glycidyl methacrylate-hyaluronic acid (GMHA): GelMA in 1:1 ratio and printed using a valve-based bioprinting technique [90]. Printing consisted of a two-step process, where a biolayer of hepatic cells was followed by a complementary layer of supporting cells (human endothelial and adipose-derived stem cells) to mimic the hepatic lobule structure. The 3D culture with three cell types showed improved morphological organization, higher liver-specific gene expression levels, and increased metabolic product secretion of hiPSC-HPCs, compared to their 2D culture. The year before, as discussed previously, Faulkner-Jones showed how iPSC at the undifferentiated state could be printed and then differentiated into hepatocyte-like cells [51]. 

### 5.4. Neural Tissue

Due to the complex physiology and limited regenerative capacity, repair of nervous system damage is challenging. Several studies have utilized bioprinting techniques to find viable solutions, which among these studies, many have shown high cell viability of neurons using 3D bioprinting technology [91]. Inkjet and microextrusion have yielded the best viability so far [60]. Joung et al. printed iPSC derived neural progenitors in 3D spinal cord constructs with promising results for disease modeling and spinal cord nervous tissue regeneration [92]. They first developed a protocol for generating oligodendrocyte progenitor cells (OPCs) from iPSC, and an additional protocol for differentiating iPSC into spinal neuronal progenitor cells (sNPCs). The two cell types were deposited as clusters of either OPCs or sNPCs embedded in a solution containing 50% Matrigel, by point-dispensing a 3D biocompatible scaffold (made either of acetoxy-based room-temperature-vulcanizing silicone, poly(ethylene glycol) diacrylate (PEGDA), or hydrogels of alginate mixed with methylcellulose (AG/MC) at different ratios) with a 200 µm center-to-center channel spacing. An outgrowth of axons and associated OPCs was detected within the printed microchannels, suggesting that sNPCs could differentiate into mature functional neurons, and OPCs into oligodendrocytes to myelinate the axons. Another study from Prof. Cook’s team gave the first example of printing undifferentiated iPSC in-situ to form self-assembled embryoid bodies, and subsequent differentiation into neurons and glia, by adding neural differentiation media, commonly used as in vitro neural inductive protocol [52]. Muller et al. used iPSC-derived neurons either alone or in co-culture with mouse iPSC-derived Schwann cells, which are cells generally present in the peripheral nervous system and can produce myelin, which surrounds neuronal axons, in 3D collagen-chitosan sponges, showing that 3D scaffold supported mature differentiation of iPSC-derived neurons. hiPSC have been also used as differentiated into neural progenitor cells (hiPSC-NPC), for the generation of organoids, by combining them with methacrylated hyaluronic acid (Me-HA) hydrogels of different stiffnesses. As a result, Wu et al. demonstrated that hiPSC-NPC undergo neural differentiation when cultured in presence of soft matrix, whereas limited neurites were observed in stiff hydrogels [93]. Additionally, hiPSC-NPC have been tested on silk-based scaffolds, pre-embedded in collagen hydrogels, showing an ability to fully differentiate into neurons within four days when cultured in vitro. Moreover, when implanted in an in vivo chicken embryo model, they were able to contribute both to formation of central and peripheral nervous system [94]. Neural stem cells were used from Hsieh et al. in 3D printed constructs aiming to repair central nervous system. Cells were first embedded in 2 thermoresponsive water-based biodegradable polyurethane dispersions hydrogels, namely PU1 and PU2, and then printed and maintained at 37 °C, in order to induce gel formation. Only cells embedded in PU2 showed great proliferation and differentiation potential, which when injected into zebrafish embryo neural injury model, could recover the function of impaired nervous system [95].

### 5.5. Skin

Skin is an ideal tissue for iPSC application since it is easily accessible, and their cells can be reprogrammed into iPSC with higher efficiency compared to other cell sources. Utilizing iPSC-MSC is a feasible strategy to apply for skin tissue repair, because of their lack of immunogenicity and reduced risk of tumorigenicity. In order to regenerate a skin wound, iPSC-MSC should undergo angiogenic and keratinogenic differentiation. Vascularization and epidermal keratinization are essential for oxygen and nutrient perfusion to the wound area, and production of renascent skin. For this purpose, iPSC-MSC are treated with basic fibroblast growth factor (bFGF) or keratinocyte growth factor (KGF) to have enhanced angiogenic and keratinogenic potential [96]. iPSC has also been used to generate endothelial cells (iEC), in 3D printed constructs for skin vascularization. Molds with vascular patterns are made from alginate and subsequently filled with human dermal fibroblasts embedded in collagen type I. Following keratinocytes addition, the construct undergoes epidermalization. At this point, alginate is dissolved by adding sodium citrate, and iEC are seeded in the same channels. Other researchers have developed vascularized skin constructs; however, these constructs are not perfusable, whereas the 3D printed micro-channels have perfusable vessels with improved endothelial barrier [97]. hiPSC have been used to develop epidermal and dermal layers by the generation of keratinocytes and fibroblasts from cord blood-derived hiPSC, respectively. By adding the epidermal layer onto the dermal layer, researchers have then been able to build a complex 3D skin organoid [98]. Materials commonly used for skin bioprinting include synthetic polymers, such as polylactic acid (PLA), PCL and Pluronic (F-127), or natural materials such as alginate, chitosan, hyaluronic acid, fibrin, and gelatin, or a combination of polyethylene glycol diacrylate and GelMA [99]. 

## 6. Conclusions

The remarkable discovery of iPSCs by Takahashi and Yamanaka has opened the door in regenerative medicine and tissue engineering to a variety of opportunities including individualized and patient-specific treatments. 3D Bioprinting of iPSC with advanced bioinks promises to develop 3D constructs with identical biofunction and architecture to the native tissue, to overcome the unmet need of a viable replacement for tissue/organ transplantation. Given the iPSC sensitivity to bioprinting parameters and conditions, particularly mechanical forces during the printing process, many hurdles should yet to be overcome to fulfill the translational promises. There is still a need for developing new biocompatible bioinks that sustain cell viability during and after printing and preserve mechanical functions over a long time. Thus far, based on existed evidence in the literature, iPSC bioprinting has shown a promising potential to address current critical challenges in regenerative medicine and been established as a versatile and scalable platform to revolutionize the regenerative medicine, disease modeling, and drug development in future. 

## Figures and Tables

**Table 1:** Compares undifferentiated iPSC literature by printing type, nozzle diameter, bioink composition, crosslinking mechanism and function.

Printing Technique	Printer	Nozzle Diameter	Bioink	Crosslinker	Cell Source	Function	Reference
Drop-on-Demand	Custom 3-axis stage	101.6 µm	1.5% w/v alginate	6% CaCl_2_	hiPSC cell lines RCi-22, RCi-50	N/A	Faulkner- Jones et al. 2015
Extrusion	Felix 3.0	40 µm	Geltrex	None	Custom made BJ fibroblasts derived hiPSC	3 germ-layers	Reid et al. 2016
	3D Bioplotter Envision TEC	200 µm	5% w/v alginate, 5% w/v carboxymethyl-chitosan, 1.5% w/v agarose	CaCl_2_	hiPSCs (source not specified)	3 germ-layers, neural tissues	Gu et al. 2017
	3D Discovery regenHu	300 µm	Nanofibrillated cellulose (NFC) alginate (60:40) NFC with HA	CaCl_2_ (for alginate) H_2_O_2_ (for HA)	Custom made A2B iPSC line, iPSC derived chondrocytes	Pluripotency, chondrocytes	Nguyen et al. 2017
	Custom-built	260 µm	2% w/v hydroxypropyl chitin (HPCH), 0-30% Matrigel	Temperature 37˚C	hiPSC from human peripheral blood mononuclear cells (hPBMCs)	Pluripotency	Li et al. 2018
Laser-assisted	Nd:YAG 1064 laser	N/A Droplet volume 0.01-1nL	1 wt% HA Matrigel	-	hiPSC from cord blood or peripheral blood-derived hiPSC line	3 germ layers	Koch et al. 2018

**Table 2:** Bioprinting of iPSC derived cells for cartilage, bone, heart, hepatic, and neural tissues. * AG/MC: Alginate mixed with metacellulose; GMHA: glycidal methacrylate-hyaluronic acid; NFC/A: alginate; NFC/HA: hyaluronic acid; OPC: oligodendrocyte progenitor cells; SNPC: induced pluripotent stem cell (iPSC)-derived spinal neuronal progenitor cells; FDM: Fused deposition manufacturing.

Tissue	Cell	Bioink	Cross-Linker	Printer	Reference
**Cartilage**	hiPSC derived chondrocytes	NFC/A* NFC/HA*	CaCl_2_	3D Discovery (regenHu, Switzerland)	*Nguyen et al. 2017*
	*iPSC source: chondrocytes*				
**Bone**	Only BM-MSC	PEG-GelMA	UV polymerization	3D inkjet printer, modified HP Deskjet 500 printer	*Gao et al. 2015*
	*(no iPSC derived)*				
**Heart**	hiPSC derived CM, SMC, EC	GelMA	^†^ Multiphoton-excitation	Custom-built multiphoton laser-scanning 3D printer	*Gao et al. 2017*
	*iPSC source: cardiac fibroblasts*				
	HUVEC and iPSC-CM	Alginate and PEG-fibrinogen hydrogel	CaCl_2_ and UV	Custom designed MPH for the simultaneous extrusion of multiple bioinks	*Maiullari et al. 2018*
	*iPSC source: mouse embryonic fibroblasts*				
	CM and EC derived from same iPSC	Decellularized omental tissue printed in supporting medium	37 ºC for 45 min	3D Discovery (RegenHU)	*Noor et al. 2019*
	*iPSC source: omental stromal cells*				
	Human skin fibroblasts	Scaffold free	--	Novogen MMX (Organova)	*Bakirci et al. 2017*
	iPSC-CM, HUVEC and NHDF	Scaffold free	--	Regenova (Cyfuse Biomedical K.K.)	*Arai et al. 2018*
**Hepatic tissue**	iPSC-HPC *iPSC source: human perinatal*	GMHA*:GelMA	UV polymerization	Custom extrusion based 3D printer	*Ma et al. 2016*
	*foreskin fibroblasts*				
**Neural tissue**	SNPC and OPC	- Matrigel as cell laden bioink - AG/MC* as supporting ink	- Temperature - CaCl_2_ or BaCl_2_	Custom microextrusion-based 3D printer	*Joung et al. 2018*
	*iPSC source:* ^†^ *UMN-X7 and UMN-3F10*				
	Neural stem cells	2 thermoresponsive water-based biodegradable polyurethane dispersions (PU1 and PU2)	Pre-crosslinking at different set of temperatures and then at 37 ºC for 4h	Self- developed FDM equipment	*Hsieh et al. 2015*
**SKIN**	iPSC derived endothelial cells	Alginate molds	CaCl_2_	^†^ Objet24 3D-Printer (Stratasys)	*Abaci et al. 2016*
	*iPSC source:* *human fibroblasts from foreskin*				

†: indicate cell are from human-induced pluripotent stem cell (hiPSC) line.

## Abbreviations

3D	Three Dimensional
bFGF	Basic fibroblast growth factor
BM-MSC	Bone marrow derived mesenchymal stem cells
CAD	Computer-aided design
CM	Cardiomyocytes
EB	Embryoid bodies
EC	Endothelial cell
ECM	Extracellular matrix
eGFP	Enhanced green fluorescent protein
EHM	Engineered heart muscle
ES	Embryonic stem cells
GelMA	Gelatin methacrylate
HA	Hyaluronic acid
hiPSC	Human induced pluripotent stem cells
hiPSC-NPC	Human induced pluripotent stem cell-derived neural progenitor cells
HPCH	Hydroxypropyl chitin
HUVEC	Human umbilical vein endothelial cells
iEC	Induced pluripotent stem cell-derived endothelial cells
iPSC	Induced pluripotent stem cells
iPSC-CM	Induced pluripotent stem cell-derived cardiomyocytes
iPSC-MSC	Induced pluripotent stem cell-derived MSC
KFG	Keratinocyte growth factor
MPH	Microfluidic printing head
MRI	Magnetic resonance imaging
mRNA	Messenger RNA
MSC	Mesenchymal stem cells
NFC	Nanofibrillated cellulose
NHDF	Normal human dermal fibroblasts
OPCs	Oligodendrocyte progenitor cells
PRP	Platelet-rich plasma
RGD	Arg-Gly-Asp
SCNT	Somatic cell nuclear transfer
SLA	Stereolithography
SMC	Smooth muscle cell
sNPCs	Spinal neuronal progenitor cells
UV	Ultraviolet
